# Evaluating a simulation-based interprofessional education activity on disaster preparedness and management among health professions students

**DOI:** 10.1186/s41077-025-00391-x

**Published:** 2025-11-28

**Authors:** Sawsan Almukdad, Aya Elhage, Lily O’Hara, Banan Mukhalalati, Mohamed Izham Ibrahim, Alla El-Awaisi

**Affiliations:** 1 Interprofessional Education Office, QU Health, Qatar University, Doha, Qatar; 2 School of Medicine and Dentistry - Public Health, Griffith University, Brisbane, Australia; 3 Department of Clinical Pharmacy and Practice, College of Pharmacy, QU Health, Qatar University, Doha, Qatar

**Keywords:** Interprofessional education, Interprofessional collaboration, Disaster preparedness, Disaster management, Simulation

## Abstract

**Background:**

Simulation-based education offers a risk-free platform to prepare future health professionals for interprofessional collaboration during high-stakes emergencies. This study involved the design, implementation, and evaluation of a disaster-focused simulation to enhance interprofessional competencies among health professions students.

**Methods:**

An interprofessional education (IPE) simulation covering the four disaster preparedness and management phases (mitigation, preparedness, response, recovery) was conducted for undergraduate health professions students. Students, assessors, and standardized patients (SPs) participated in the evaluation. Data on interprofessional competencies were collected from students using the Team’s Perception of Collaborative Care Questionnaire, from assessors using the Modified McMaster-Ottawa Scale, and from SPs using the Standardized Patient Team Evaluation Instrument. Descriptive statistics were used to summarize study variables. Paired sample t-tests were conducted to compare score differences between assessors. Learning curve across cases were tested using one-way repeated measures ANOVA, and associations between global scores and demographic variables were analyzed using t-test or ANOVA, as appropriate.

**Results:**

Thirty-three students, 13 assessors, and 8 SPs participated in the evaluation. response rates were 33.3% (students), 92.9% (assessors), and 100% (SPs). Students self-reported positive perceptions of teamwork in the activity, with over 90% agreement across all domains. Assessors’ ratings for the response phase corroborated these findings, with over 80% of students scoring at or above expectations in all domains. SPs’ evaluations were also high, with 70% agreeing that students demonstrated positive interprofessional practice behaviors. For the diabetic ketoacidosis case, teams’ global performance scores were calculated as the mean of the two assessors’ ratings. Students with prior IPE experience (M = 2.42, 95% CI: 2.24–2.60) and those who had completed a prior practice placement (M = 2.48, 95% CI: 2.30–2.65) performed significantly better than students without IPE experience (M = 2.06, 95% CI: 1.80–2.33) or a prior practice placement (M = 2.12, 95% CI: 1.86–2.37). While not statistically significant, a trend towards improved performance across cases in the response phase suggested a learning curve effect.

**Conclusions:**

Simulation-based IPE can strengthen interprofessional competencies for disaster preparedness and management, with greatest benefit when preceded by other IPE activities and clinical placements.

**Supplementary Information:**

The online version contains supplementary material available at 10.1186/s41077-025-00391-x.

## Introduction

Interprofessional education (IPE) is an approach that aims to prepare health professions students to learn with, from, and about each other [1]. The World Health Organization (WHO), in its *Framework for Action on Interprofessional Education and Collaborative Practice,* emphasizes the importance of integrating IPE into health profession curricula to build a workforce capable of addressing today’s complex health and social care challenges through effective collaboration [2]. Collaboration among health professionals offers numerous benefits, including improved quality of care and patient safety [3], enhanced patient outcomes [4, 5], and greater satisfaction among both patients and health professionals [6, 7].

Simulation-based education is widely used to teach technical and clinical skills to health professions students [8]. In simulation-based IPE, students from different professions collaborate during simulated scenarios, enhancing learning and preparing them for effective teamwork in real-world settings [9–11]. In these simulations, students work in interprofessional teams to develop care plans, manage patient scenarios, and make decisions in a safe environment [12–14]. Simulation-based IPE has demonstrated beneficial outcomes, such as improved self-reported knowledge, confidence, and collaborative abilities [11].

Global health challenges, including natural and anthropogenic disasters, require a united and collaborative response. Given the increasing frequency and severity of these disasters [15, 16], a collaborative approach to disaster preparedness and management is critical [17, 18]. However, poor coordination among health professionals during disasters remains a major obstacle, hindering the delivery of critical health and social care services and exposing significant gaps in collaboration [19–21]. This highlights the importance of developing strategies to enhance collaboration during disaster preparedness and response efforts [22].

Simulation-based education provides an immersive, experiential platform for preparing health professionals to navigate the complexities of real-world emergencies. In disaster scenarios, where rapid decision-making, coordination under pressure, and prioritization of limited resources are critical, simulation enables learners to rehearse clinical and collaborative actions in a controlled, risk-free environment [23, 24]. Simulation, combined with interprofessional collaboration, provides a unique opportunity to strengthen both individual competencies and team dynamics essential for effective disaster response [13]. Although some studies have examined IPE in disaster preparedness and management [25–31], there is limited literature describing the design, delivery, and evaluation of simulation-based IPE activities. To address this gap, we designed and delivered a simulation-based IPE activity focused on disaster preparedness and management, and evaluated the impact on health professions students participating in the activity, incorporating perspectives from students, assessors, and standardized patients (SPs).

## Methods

### Format

A simulation-based IPE activity was designed for undergraduate health professions students addressing the four phases of disaster preparedness and management: mitigation, preparedness, response, and recovery [32]. The activity was part of the Ninth Annual Qatar IPE Student Forum, an extracurricular event focused on disaster preparedness and management. The objectives of the simulation activity were for students to engage in interprofessional teamwork by effectively communicating roles, responsibilities, and information sharing within a simulated disaster scenario; apply individual professional knowledge to contribute to comprehensive disaster response actions; and demonstrate the ability to make timely and informed decisions during a disaster scenario, incorporating input from diverse professionals to optimize client care.

### Participants

The intended participant numbers were 40 undergraduate health professions students from Qatar University (QU) dental medicine, health sciences, medicine, nursing, and pharmacy programs and nursing students from the University of Calgary-Qatar, 16 assessors, and 8 SPs. These were the maximum number of participants that could feasibly be accommodated in the simulation setting. All participants were recruited through convenience sampling. Inclusion criteria for students, assessors, and SPs were 18 years or older, affiliated with any health profession college or university in Qatar (for students and assessors), and fluent in English or Arabic.

### Activity description

A mandatory in-person orientation session for assessors and SPs was held one week before the activity including a presentation outlining the activity description and timeline, a video tutorial on how to use the Modified McMaster-Ottawa Scale, and role-playing between assessors and SPs to practice using the scales. The training session aimed to enhance assessors’ and SPs’ confidence in evaluating interprofessional teams and promote consistency in their assessments. One week before the activity, students were provided with pre-reading materials. An online orientation session was conducted three days prior to the activity to familiarize students with the activity and its objectives. While attendance was highly encouraged, it was voluntary and thus not formally recorded. The session covered the disaster scenario, structure and flow of the activity, expected tasks at each station, and duration of each phase. Stratified random distribution was used to create interprofessional teams of three to five members, ensuring representation from different health professions in each team.

On the day of the activity, students, assessors, and SPs gathered in an assembly room. The students were then grouped into their pre-assigned teams. The activity began with two static stations (not involving interaction with assessors or SPs) on the mitigation and preparedness phases, each lasting 15 min. At the stations, computer tablets provided students the information needed to complete the tasks. After the mitigation and preparedness phases, the response phase started with a simulated bomb alarm, alerting participants to an emergency. The disaster scenario was an explosion in a public space, requiring students to perform rapid triage and provide immediate care while coordinating across professions.

During the response phase, teams rotated through four cases, each involving an SP with a health issue relevant to a disaster, including diabetic ketoacidosis, post-traumatic stress disorder, chemical exposure, and an infectious disease. For each case, students conducted assessments, developed management plans, and delivered interprofessional care. Each case was observed by two assessors and lasted 20 min. After the completion of all four cases, students spent 15 min at the static recovery phase station. The description of the four phases, including the tasks assigned to students, is provided in Supplementary Box 1. A detailed description of the activity structure and disaster scenario is provided in the supplementary materials, including the organization and sequence of stations (Supplementary Fig. 1), composition of teams (Supplementary Table 1), and the four cases in the response phase (Supplementary Table 2).

### Instruments

#### Student self-evaluation

The Team’s Perception of Collaborative Care Questionnaire was completed by students individually to assess their perceptions of the team-based collaborative care they provided during the simulation [33]. The instrument consists of 20 items rated on a five-point Likert-type scale (1 = strongly disagree, 3 = neither agree nor disagree, 5 = strongly agree), evaluating five components: values and ethics, roles and responsibilities, communication, teamwork, and self-evaluation.

#### Assessor evaluation

The modified McMaster-Ottawa Scale was used by assessors to evaluate individual student performance and interprofessional team functioning [34, 35]. This three-point scale (1 = below expected, 2 = at expected, 3 = above expected) incorporates standardized behavioral anchors to guide assessors in evaluating six IPE competencies: communication, collaboration, roles and responsibilities, collaborative patient-centered approach, conflict management, and team functioning. It also included a global rating score, offering a single rating of both the individual’s and team’s performance, based on the combined ratings across all six competencies. A final open-ended question asked: “Why did you choose this rating for the overall global rating?”.

#### Standardized patient team evaluation

The Standardized Patient Team Evaluation Questionnaire was used to assess the SP’s perspective on the interprofessional team functioning [33]. This instrument comprised 10 items rated on a five-point Likert-type scale (1 = strongly disagree, 3 = neither agree nor disagree, 5 = strongly agree).

### Data collection process

The research team provided assessors and SPs with hard copies of the evaluation instruments at the start of the day and collected completed instruments immediately after the activity. Following the activity, students received an email with a link to the Team’s Perception of Collaborative Care Questionnaire hosted online on the SurveyMonkey® platform. Students were given up to one week after the activity to complete their questionnaires, facilitating a higher response rate.

### Data analysis

Statistical analysis was performed using Stata/SE 18 (StataCorp LLC, USA). Descriptive statistics (means, standard deviations, frequencies, percentages) summarized study variables. Paired sample t-tests were used to examine score differences between the two assessors. Following this, individual student and team scores were calculated as the average of the scores assigned by both assessors for each case. To investigate the learning curve effect, a one-way repeated measure analysis of variance (ANOVA) test was conducted to compare scores across the four cases, followed by Tukey’s HSD test for pairwise comparisons if significant. Associations between the individual student mean global score and student demographic variables were assessed using the independent t-test or one-way ANOVA depending on the number of categories in the demographic variable. The mean score of the global score for team performance was calculated using the average of the two assessors’ ratings. P-values less than 0.05 were considered statistically significant. Narrative synthesis of the brief qualitative responses to the open-ended question for the assessors was performed to contextualize the quantitative results.

### Ethics consideration

All information was collected anonymously from participants, who were assured that their details would remain confidential and used solely for research purposes. Informed written consent was obtained from all participants. The study adhered to the 1964 Helsinki Declaration and was approved by the QU Institutional Review Board (QU-IRB 1901-EA/23).

## Results

### Participants

Response rates were 33.3% for students (11 of 33 participants), 92.9% for assessors (13 of 14 participants), and 100% for SPs (8 of 8 participants). Demographic characteristics of participants are provided in Table 1. Over 90% of students were female, and more than two-thirds were in their third year of study. Approximately 85% participated in previous IPE activities, and 60% completed a practice placement course or rotation. Over 90% of assessors were female, with 38% from QU College of Pharmacy. Around 70% of assessors had no prior experience in disaster preparedness and management, although more than 50% reported expertise in IPE. Eighty-eight percent of the SPs were female, and 75% had no prior IPE experience.

**Table 1:** Characteristics of participants

Characteristic	Number (%)
Students (*N* = 33)	
Age, mean (SD) (*N* = 33)	21.75 (1.50)
Age group (*N* = 33)	
< 21 years	16 (48.48)
≥ 21 years	17 (51.52)
Gender (*N* = 33)	
Female	31 (93.94)
Male	2 (6.06)
Nationality (*N* = 33)	
Bahrain	1 (3.03)
Bangladesh	5 (15.15)
Egypt	1 (3.03)
India	1 (3.03)
Iran	2 (6.06)
Jordan	2 (6.06)
Libya	2 (6.06)
Oman	1 (3.03)
Pakistan	4 (12.12)
Palestine	1 (3.03)
Philippines	8 (24.24)
Qatar	2 (6.06)
Sudan	1 (3.03)
Syria	2 (6.06)
Student profession (*N* = 33)	
Biomedical	2 (6.06)
Dental Medicine	1 (3.03)
Medicine	6 (18.18)
Nursing	14 (42.42)
Human Nutrition	1 (3.03)
Pharmacy	5 (15.15)
Physical Therapy	3 (9.09)
Public Health	1 (3.03)
Professional year (*N* = 33)	
Year 1	4 (12.12)
Year 2	9 (27.27)
Year 3	13 (39.39)
Year 4	5 (15.15)
Year 5	2 (6.06)
Prior participation in IPE activities (*N* = 33)	
No	8 (75.76)
Yes	25 (24.24)
Prior experiences related to disaster preparedness and management (*N* = 33)	
No	28 (84.85)
Yes	5 (15.15)
Prior completion of a practice placement course/rotation	
No	13 (39.39)
Yes	20 (60.61)
Assessors (*N* = 13)	
Age, mean (SD) (*N* = 13)	33.85 (10.04)
Gender (*N* = 13)	
Male	1 (7.69)
Female	12 (92.31)
Nationality (*N* = 12)	
Algeria	1 (8.33)
Australia	1 (8.33)
Canada	4 (33.33)
Egypt	2 (16.67)
India	1 (8.33)
Jordan	1 (8.33)
Lebanon	1 (8.33)
USA	1 (8.33)
Assessor profession (*N* = 13)	
Biomedical	1 (7.69)
Nursing	1 (7.69)
Nutrition	1 (7.69)
Pharmacy	5 (38.46)
Pharmacy Technician	1 (7.69)
Physical Therapy	2 (15.38)
Public Health	2 (15.38)
Prior experiences related to IPE (*N* = 13)	
Yes	6 (46.15)
No	7 (53.85)
Prior experiences related to disaster preparedness and management (*N* = 13)	
Yes	4 (30.77)
No	9 (69.23)
Standardized patients (*N* = 8)	
Age, mean (SD) (*N* = 6)	45.33 (16.90)
Gender (*N *= 8)	
Male	1 (12.50)
emale	7 (87.50)
Nationality (*N* = 8)	
India	1 (12.50)
Mauritius	1 (12.50)
Philippines	2 (25.00)
South Africa	1 (12.50)
Sudan	2 (25.00)
United Kingdom	1 (12.50)
Prior experiences related to IPE (*N* = 8)	
Yes	2 (25.00)
No	6 (75.00)

### Student self-evaluation

Students’ self-reported perceptions of their interprofessional teamwork are presented in Table 2. In the values and ethics domain, 91% agreed or strongly agreed that they had worked well with other health professions, and 100% had established trust, focused on patient safety, and managed ethical dilemmas effectively. Regarding roles and responsibilities, 91% agreed or strongly agreed that they had described their roles clearly, 100% had engaged other professionals and utilized their knowledge and skills, and 81% had explained others’ roles accurately. Regarding communication skills, 91% agreed or strongly agreed that they had conveyed information clearly, and 100% had contributed to care decisions and demonstrated active listening and respectful conflict interaction. For teamwork, 91% agreed or strongly agreed that they had engaged in collaborative problem-solving, 82% had demonstrated leadership, and 100% had accepted responsibility and performed effectively in various roles. All students agreed or strongly agreed that they had acted ethically, identified strengths and weaknesses, recognized the impact of effective communication, and engaged in problem-solving.

**Table 2:** Students’ perception of team performance in providing collaborative care

		**Number (%)**				
**Items**	**Number responded**	**Strongly** **agree**	**Agree**	**Neither agree nor** **disagree**	**Disagree**	**Strongly** **disagree**
**Values/Ethics Components**						
1. Worked well with and respected the values and expertise of other health professionals	11	5 (45.45)	5 (45.45)	0 (0.00)	1 (9.09)	0 (0.00)
2. Established trust and rapport with patients, families and team members	11	3 (27.27)	8 (72.73)	0 (0.00)	0 (0.00)	0 (0.00)
3. Focused on quality of care and patient safety	11	5 (45.45)	6 (54.55)	0 (0.00)	0 (0.00)	0 (0.00)
4. Managed ethical dilemmas in interprofessional care situations	11	2 (18.18)	9 (81.82)	0 (0.00)	0 (0.00)	0 (0.00)
**Roles and Responsibilities Components**						
5. Described own roles and responsibilities clearly to patients and team members	11	5 (45.45)	5 (45.45)	0 (0.00)	1 (9.09)	0 (0.00)
6. Engaged other healthcare professionals with different expertise in the evaluation and treatment process	11	6 (54.55)	5 (45.45)	0 (0.00)	0 (0.00)	0 (0.00)
7. Explained roles and responsibilities of other providers accurately	11	5 (45.45)	4 (36.36)	1 (9.09)	1 (9.09)	0 (0.00)
8. Utilized knowledge, skills, and abilities of team members to optimize patient care	11	4 (36.36)	7 (63.64)	0 (0.00)	0 (0.00)	0 (0.00)
**Communication Components**						
9. Communicated information in a form that is understandable to non-healthcare professionals	11	4 (36.36)	6 (54.55)	0 (0.00)	0 (0.00)	1 (9.09)
10. Contributed to conversations leading to care decisions	11	5 (45.45)	6 (54.55)	0 (0.00)	0 (0.00)	0 (0.00)
11. Demonstrated active listening, encouraged ideas, and provided timely, constructive feedback	11	4 (36.36)	7 (63.64)	0 (0.00)	0 (0.00)	0 (0.00)
12. Interacted in a respectful manner when dealing with conflict	11	6 (54.55)	5 (45.45)	0 (0.00)	0 (0.00)	0 (0.00)
**Teamwork Components**						
13. Engaged in collaborative problem solving among healthcare professionals	11	5 (45.45)	5 (45.45)	0 (0.00)	1 (9.09)	0 (0.00)
14. Demonstrated leadership practices, which facilitated effective teamwork	11	3 (27.27)	6 (54.55)	1 (9.09)	1 (9.09)	0 (0.00)
15. Accepted responsibility for teamwork outcomes	11	6 (54.55)	5 (45.45)	0 (0.00)	0 (0.00)	0 (0.00)
16. Performed effectively as a team by participating in a variety of roles	11	6 (54.55)	5 (45.45)	0 (0.00)	0 (0.00)	0 (0.00)
**Self-Evaluation Components**						
17. You acted ethically with honesty and integrity	11	9 (81.82)	2 (18.18)	0 (0.00)	0 (0.00)	0 (0.00)
18. You identified your strengths and weaknesses	11	9 (81.82)	2 (18.18)	0 (0.00)	0 (0.00)	0 (0.00)
19. You recognized the impact of effective communication skills on patient care	11	7 (63.64)	4 (36.36)	0 (0.00)	0 (0.00)	0 (0.00)
20. You participated in collaborative problem solving	11	6 (54.55)	5 (45.45)	0 (0.00)	0 (0.00)	0 (0.00)

### Assessor evaluation

Statistical analysis using paired sample t-tests showed no significant differences between the two assessors in individual student scores or team scores for any case (Table 3). Therefore, individual student and team scores were determined by calculating the average of the scores given by the two assessors. The assessors’ ratings of individual student performance are presented in Table 4. Over 80% of students were rated as expected or above expected across all domains. In the communication skills and collaboration domains, almost half of the students were rated as expected (47% and 45%, respectively) and over a third were rated as above expected. Positive performances were also observed in role definition, collaborative patient-family centered approach, and conflict management. In the team functioning domain, 43% of students were rated as expected, and 39% as above expected. The global rating of individual performance aligned with these findings, with 57% rated as expected and 28% rated as above expected.

**Table 3:** Differences between individuals and team scores by assessor and by case

	**Individual rating scale**									
	Track 1					Track 2				
	Assessor 1	Assessor 2				Assessor 3	Assessor 4			
Case title	Mean (SD)		Mean difference	t-test	p-value	Mean (SD)		Mean difference	t-test	p-value
Diabetic ketoacidosis	2.08 (0.49)	2.31 (0.48)	−0.23	−1.39	0.190	2.25 (0.64)	2.55 (0.51)	−0.30	−1.83	0.083
Posttraumatic stress disorder	2.31 (0.85)	2.15 (0.38)	0.15	0.51	0.613	2.10 (0.64)	2.00 (0.69)	0.15	1.83	0.083
Chemical exposure	1.85 (0.69)	1.85 (0.69)	0.00	0.00	1.000	Not applicable^a^				
Infectious disease outbreak	2.08 (0.76)	2.23 (0.73)	−0.15	−1.00	0.337	Not applicable^a^				
	**Team rating scale**									
	Track 1					Track 2				
	Assessor 1	Assessor 2				Assessor 3	Assessor 4			
Case title	Mean (SD)		Mean difference	t-test	p-value	Mean (SD)		Mean difference	t-test	p-value
Diabetic ketoacidosis	2.00 (0.82)	2.25 (0.50)	−0.25	−1.00	0.391	2.25 (0.96)	2.00 (0.00)	0.25	0.52	0.638
Posttraumatic stress disorder	2.50 (0.58)	2.50 (0.58)	0.00	0.00	1.000	2.00 (0.82)	1.75 (0.96)	0.25	1.00	0.391
Chemical exposure	2.00 (0.82)	2.00 (0.82)	0.00	0.00	1.000	Not applicable^a^				
Infectious disease outbreak	2.25 (0.96)	2.50 (0.58)	−0.25	−1.00	0.391	Not applicable^a^				

*SD *Standard deviation

^a^Two assessors did not attend on the day of the activity, and one assessor did not provide a consent form

**Table 4:** Individual rating for assessor pairs across all cases

		**Number (%)**		
**Competencies**	**Total responses**^**a**^	**Below expected**	**At expected**	**Above expected**
**Communication (with other team members)** Assertive communication Respectful communication Effective communication	204	38 (18.63)	96 (47.06)	70 (34.31)
**Collaboration** Establishes collaborative relationships Integration of perspectives Ensures shared information	204	35 (17.16)	92 (45.10)	77 (37.75)
**Roles and responsibilities** Describes roles and responsibilities Shares knowledge with others; Accepts accountability	204	27 (13.24)	99 (48.53)	78 (38.24)
**Collaborative patient-family centered approach** Seeks input from patient and family Shares with patient and family Advocates for patient and family	204	29 (14.22)	99 (48.53)	76 (37.25)
**Conflict management/resolution** Demonstrates active listening Respectful of different perspectives Works with others to prevent conflict	204	25 (12.25)	112 (54.90)	67 (32.84)
**Team functioning** Evaluates team function and dynamics Contributes effectively Demonstrates shared leadership	204	38 (18.63)	87 (42.65)	79 (38.73)
**Global rating – score** Provide a single rating of the individual’s performance	202	30 (14.85)	115 (56.93)	57 (28.22)

^a^We had a total of 13 assessors evaluating teams, with each team consisting of 3–5 students

The assessors’ ratings of team performance are presented in Table 5. The results indicate that more than two-thirds of teams were rated as expected or above expected across all domains. Over 40% of teams were rated as above expected in communication, collaboration, collaborative patient-family-centered approach, and conflict management. Approximately one-third of teams were rated as above expected in team functioning. Almost 80% of teams were rated as at or above expected for global team performance.

**Table 5:** Team rating for assessor pairs across all cases

		**Number (%)**		
**Competencies**	**Total responses**^**a**^	**Below expected**	**At expected**	**Above expected**
**Communication (of team with patient)** Assertive communication Respectful communication Effective communication	52	8 (15.38)	22 (42.31)	22 (42.31)
**Collaboration** Establishes collaborative relationships Integration of perspectives Ensures shared information	52	7 (13.46)	24 (46.15)	21 (40.38)
**Roles and responsibilities** Describes roles and responsibilities Shares knowledge with others; Accepts accountability	52	8 (15.38)	27 (51.92)	17 (32.69)
**Collaborative patient-family centered approach** Seeks input from patient and family Shares with patient and family Advocates for patient and family	52	12 (23.08)	16 (30.77)	24 (46.15)
**Conflict management/resolution** Demonstrates active listening Respectful of different perspectives Works with others to prevent conflict	52	9 (17.31)	21 (40.38)	22 (42.31)
**Team functioning** Evaluates team function and dynamics Contributes effectively Demonstrates shared leadership	51	11 (21.57)	18 (35.29)	22 (43.14)
**Global rating – score** Provide a single rating of the team’s performance	51	9 (17.65)	27 (52.94)	15 (29.41)

^a^We had a total of 13 assessors, each evaluating 4 teams

**Fig. 1:**
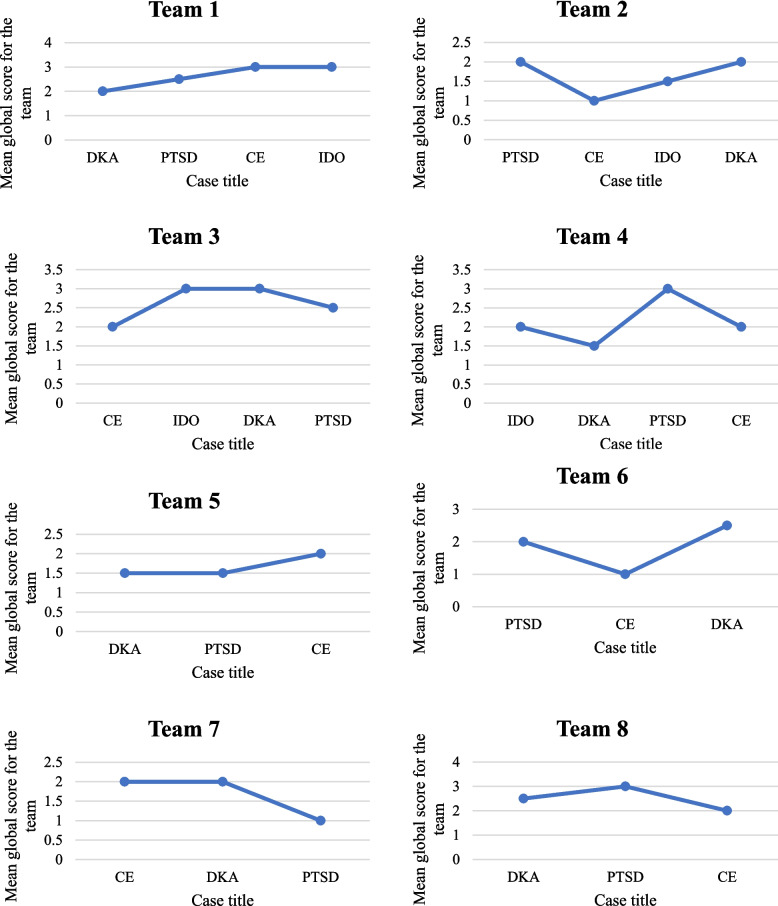
Learning curve effect, examining the potential improvement between the first, second, third, and fourth cases for the eight teams. Abbreviations: CE, Chemical exposure; DKA, Diabetic ketoacidosis; IDO, Infectious disease outbreak; PTSD, Posttraumatic stress disorder

The learning curve effect for the eight teams is illustrated in Fig. 1. While some teams demonstrated increases in mean global scores as students progressed through the four cases, this pattern was not consistent across all teams. Overall, the ANOVA indicated that these differences were not statistically significant (F(3, 67) = 2.64, p = 0.0567). However, post-hoc analysis revealed differences in the diabetic ketoacidosis case, whereby students who had previously participated in IPE (M = 2.42, 95% CI: 2.24–2.60) scored significantly higher than students who had not participated in IPE (M = 2.06, 95% CI: 1.80–2.33), and students who had completed a practice placement (M = 2.48, 95% CI: 2.30–2.65 scored significantly higher than those who had not (M = 2.12, 95% CI: 1.86–2.37). There were no other significant associations between student scores and demographic variables. Gender was excluded from the association analysis due to the small number of male students (n = 2). No significant associations were found in the other three cases.

Qualitative insights from the assessors into the factors influencing overall team performance scores are outlined in Supplementary Table 4. Assessors felt that teams with below-expectation ratings commonly faced challenges such as limited patient involvement, unequal participation among team members, communication breakdowns, and a lack of collaboration. Additionally, these teams occasionally demonstrated conflicting viewpoints among members and used medical terminology when communicating with patients. In contrast, assessors felt that teams with above-expectation ratings demonstrated several strengths, including a patient-centered approach that emphasized engagement, appropriate care delivery, effective teamwork, and active listening skills. They also exhibited clear role delineation before patient involvement, efficient allocation of responsibilities, and shared decision-making.

### Standardized patient team evaluation

The SPs’ evaluations of student team performances are outlined in Table 6. Three-quarters of the SPs agreed or strongly agreed that student teams demonstrated all interprofessional practice statements. More than 80% of SPs agreed or strongly agreed that students supported one another, provided information understandably, and gained their trust. Around 75% of SPs agreed or strongly agreed that students demonstrated effective communication among team members, teamwork, role clarity, discussions about care plans, collaborative planning, and leadership skills. Furthermore, 90% of SPs agreed or strongly agreed that students assisted one another with their care.

**Table 6:** Standardized patient team evaluation across the four cases

		**Number (%)**				
**Items**	**Total responses**^**a**^	**Strongly** **agree**	**Agree**	**Neither agree nor** **disagree**	**Disagree**	**Strongly** **disagree**
1. There appeared to be good communication among the team members	31	10 (32.26)	13 (41.94)	5 (16.13)	3 (9.68)	0 (0.00)
2. Team members were supportive and respectful to other team members	31	17 (54.84)	9 (29.03)	5 (16.13)	0 (0.00)	0 (0.00)
3. The team members provided information to me in a manner that was easy to understand	31	12 (38.71)	15 (48.39)	2 (6.45)	2 (6.45)	0 (0.00)
4. The team gained my trust and acted ethically	30	14 (46.67)	11 (36.67)	3 (10.00)	2 (6.67)	0 (0.00)
5. The team worked well together to coordinate my care	31	12 (38.71)	12 (38.71)	3 (9.68)	3 (9.68)	1 (3.23)
6. Team members made it clear to me what their roles were	31	14 (45.16)	9 (29.03)	3 (9.68)	3 (9.68)	2 (6.45)
7. Team members helped one another with my care	30	14 (46.67)	13 (43.33)	2 (6.67)	1 (3.33)	0 (0.00)
8. The team members discussed with me my care and supported my decisions about that care	31	13 (41.94)	11 (35.48)	5 (16.13)	2 (6.45)	0 (0.00)
9. The team members collaborated and agreed with my plan of care	31	13 (41.94)	11 (35.48)	6 (19.35)	1 (3.23)	0 (0.0)
10. The team members demonstrated leadership practices that led to effective teamwork	31	13 (41.94)	10 (32.26)	6 (19.35)	1 (3.23)	1 (3.23)

^a^We had a total of 8 standardized patients, each evaluating 4 teams

## Discussion

This study provides the first description of the design and delivery of a simulation-based IPE activity focused on disaster preparedness and management, and evaluation of the impact on health professions students’ performance from the perspectives of participating students, assessors, and SPs. Students reported positive perceptions of their interprofessional teamwork during the activity with respect to ethics, roles and responsibilities, communication, and teamwork. This suggests that a well-designed learning environment can foster confidence and competence in interprofessional collaboration, aligning with prior research on students’ positive attitudes toward team-based care [36, 37]. Participating in collaborative IPE experiences enhances students’ self-confidence, understanding of teamwork principles, and ability to work effectively in teams [38, 39].

There were no significant differences between the two assessors in individual student scores or team scores for any case, indicating that the evaluations were consistent and reliable. Furthermore, the assessors’ ratings aligned closely with the students’ self-assessments, with over 80% of students strongly agreeing or agreeing with statements across all domains of the Team’s Perception of the Collaborative Care Instrument. These positive findings were reinforced by evaluations from SPs, with more than 70% of SPs strongly agreeing or agreeing with the statements in all domains of the Standardized Patient Team Evaluation instrument. This alignment supports the validity of the results and demonstrates that assessors and SPs were well equipped to evaluate students’ interprofessional competencies.

A pattern of student performance improvement across the cases was observed, even though the learning curve analysis was not statistically significant (Fig. 1). This suggests that repeated exposure to different cases may enhance interprofessional skills such as teamwork, communication, and problem-solving [40]. Findings are consistent with experiential learning theory, whereby repeated, scaffolded exposure and structured debriefing consolidate team communication and role clarity [41–43]. In the diabetic ketoacidosis case, performance was significantly stronger among students with prior IPE experience and those who had completed a practice placement. This highlights the importance of both IPE experiences and practice placements and the necessity to integrate them into curricula to prepare future health professionals for collaborative practice [7, 44].

Assessors’ qualitative comments indicated that underperforming teams struggled with challenges, for example, ineffective communication, unequal participation, and a lack of teamwork. These challenges reflect known barriers to effective interprofessional collaboration and highlight areas for targeted strategies in future training programs [45–47]. High-performing teams, on the other hand, showed strengths in patient-centered care, clear role definition, and shared decision-making, highlighting the significance of these elements in effective interprofessional practice [45–47].

The study had several strengths and limitations. To our knowledge, this is the first study to evaluate a simulation-based IPE activity integrating perspectives from students, assessors, and SPs, offering a comprehensive evaluation of both individual and team performance. The use of validated assessment instruments enhanced the reliability and validity of the findings. The study achieved a high response rate from both assessors and SPs, strengthening the robustness of the collected data. However, the study also has limitations. The student response rate was low, possibly due to the length of the questionnaire. No additional demographic or performance-related data were collected to allow comparison between responders and non-responders. As this was a pilot study, no formal power analysis was conducted, and the number of participants was based on logistical consideration of the venue capacity. The low student response rate limited the ability to detect small to moderate effects, particularly in subgroup analyses. Although the positive assessor ratings, student self-assessments, and SP evaluations are encouraging, it is impossible to rule out social desirability bias, which could have resulted from participants feeling under pressure to respond positively. Finally, participants were recruited through convenience sampling, which may have introduced sampling bias and limited the generalizability of the findings. The predominance of female participants (93.9%), whilst a reflection of current enrollment statistics, further limits the generalizability of the study findings to all students.

### Recommendations

Given the observed improvements in student performance through repeated practice and active engagement, incorporating multiple case-based simulations can enhance interprofessional skills. Additionally, students with prior IPE experience and those who had completed a practice placement demonstrated stronger performance, highlighting the need to integrate structured IPE activities and clinical placements into health professions curricula. Embedding these experiences early in training programs will better prepare future health professionals for effective collaborative practice. Triangulating student, assessor, and standardized-patient perspectives is feasible and yields a rounded view of performance, but future evaluations should report inter-rater reliability and complement ordinal ratings with observable behavior measures. In addition, longitudinal studies are recommended to determine whether gains persist and translate to clinical placements and early practice.

### Implications

A well-designed simulation-based IPE activity that evaluates students’ performance in disaster preparedness and management, integrating multiple perspectives from students, assessors, and SPs, is essential for enhancing competence in interprofessional collaboration. It enables health programs to better equip students for the challenges of health practice, ultimately leading to improved patient outcomes. Future efforts should focus on refining these educational approaches, thereby increasing the impact and reach of IPE initiatives. In parallel, regular joint exercises with local services including public health and primary care partners can align student learning with regional disaster plans and enhance system preparedness.

## Conclusions

The study’s findings support the value of IPE in strengthening disaster preparedness and management skills. The strong agreement across student self-evaluations, assessor ratings, and SPs feedback highlights the credibility of the findings. Simulation-based IPE has potential to develop student interprofessional competencies in disaster preparedness and management and may be most effective after students have experienced other IPE activities and completed a practice placement.

## Supplementary Information

Additional file 1.

## Data Availability

All relevant data are presented in the manuscript and its supplementary material file.
