# Supplementary material for: Evaluating a simulation-based interprofessional education activity on disaster preparedness and management among health professions students
Source: Adv Simul (Lond). 2025 Nov 28;10:62. doi: 10.1186/s41077-025-00391-x (PMC12664206; doi:10.1186/s41077-025-00391-x)
Supplement: Supplementary file 1 — Additional file 1. [file 41077_2025_391_MOESM1_ESM.docx]

**Supplementary Material**

**Disaster Preparedness and Management through Interprofessional Education: A Simulation-Based Study among Health Professions Students**

## **Supplementary Box 1. Details of the four phases of disaster preparedness and management.**

**Station 1: Mitigation (15 minutes)**

- Before the session, all students were required to review the guidelines and protocols and come prepared to discuss them at mitigation stage.
- No standardized patients were required.

Tasks:

1. Discuss the available protocols and guidelines related to disaster management that are available to you and identify their strengths and weaknesses.
2. Reflect on how these documents are related to a bombing disaster in a war situation.

**Station 2: Preparedness (15 minutes)**

- In preparation for potential bombing, students should participate in an exercise where they discuss and develop plans for effective response strategies. Students worked on understanding their roles and responsibilities, as well as creating a plan for the management of bombing during a war.
- No standardized patients were required.

Tasks:

1. Develop an emergency response plan for health professionals.
2. Develop strategies for community education and awareness to reduce vulnerability.

**Station 3: Response (Four cases will occur simultaneously for 20 minutes for each case)**

The immediate aftermath requires a coordinated and dynamic response. Students will navigate through different stations, making critical decisions to address the diverse injuries and health related issues resulting from the bombing.

Tasks:

1. Assess and treat standardized patients with different health issues.
2. Collaborate with other health professions students.

**Station 4: Recovery (15 minutes)**

- As the city enters the recovery phase, students focused on restoring health and social services, addressing long-term physical and mental health challenges, and rebuilding the community.
- No standardized patients were required.

Tasks:

1. Each student briefly will discuss how they would contribute to the recovery efforts, considering their unique skills and perspectives.
2. Create a plan that includes strategies to promote community awareness related to cases in the response stations.
3. As a team, present specific challenges related to the recovery phase (e.g., shortage of medical supplies). Afterwards, in their groups, students brainstorm quick, practical solutions that involve collaboration between different health professions.
4. During the debriefing session, share your reflections on the entire activity.

## **Supplementary Figure 1. Organization and sequencing of the stations.**

**Station 1: Mitigation**

Labs Involved: Lab 1 and Lab 2

Group Distribution: Each lab will consist of 4 groups with 5 students per group

Duration: 15 minutes

**Station 2: Preparedness**

Labs Involved: Lab 1 and Lab 2

Group Distribution: Each lab will consist of 4 groups with 5 students per group

Duration: 15 minutes

**Station 3: Response**

Activity: Response scenarios conducted simultaneously

Duration: 20 minutes per case

*Diabetic Ketoacidosis - Lab 1*

Group Distribution: 2 groups of 5 students each

Facilitation: 2 assessors per group

Standardized Patients (SPs): 1 SP per group

*Posttraumatic Stress Disorder - Lab 2*

Group Distribution: 2 groups of 5 students each

Facilitation: 2 assessors per group

Standardized Patients (SPs): 1 SP per group

*Chemical Exposure - Lab 3*

Group Distribution: 2 groups of 5 students each

Facilitation: 2 assessors per group

Standardized Patients (SPs): 1 SP per group

*Infectious Disease Outbreak - Lab 4*

Group Distribution: 2 groups of 5 students each

Facilitation: 2 assessors per group

Standardized Patients (SPs): 1 SP per group

**Station 4: Recovery**

Labs Involved: Lab 1 and Lab 2

Group Distribution: Each lab will consist of 4 groups with 5 students per group

Duration: 15 minutes

5-minutes break

5-minutes break

5-minutes break

5-minutes break

| Team 1 (N=3) | Nursing  Medicine  Physical Therapy |
| --- | --- |
| Team 2 (N=4) | Dental Medicine  Human Nutrition  Pharmacy  Nursing |
| Team 3 (N=3) | Medicine  Nursing_A  Nursing_C |
| Team 4 (N=3) | Pharmacy  Nursing  Public Health |
| Team 5 (N=5) | Nursing_A  Biomedical  Nursing_B  Nursing_C  Medicine |
| Team 6 (N=5) | Physical Therapy  Medicine  Nursing_A  Pharmacy  Nursing_B |
| Team 7 (N=5) | Medicine  Pharmacy  Nursing_A  Nursing_B  Physical Therapy |
| Team 8 (N=5) | Nursing_A  Nursing_B  Medicine  Pharmacy  Biomedical |

## **Supplementary Table 1. Distribution of students who attended the activity among the teams.**

## **Supplementary Table 2. Cases during the response phase.**

| **Case number** | **Case title** | **Case description** | **Student task** |
| --- | --- | --- | --- |
| Case 1 | Diabetic ketoacidosis | A standardized patient presents with diabetic ketoacidosis and reveals that they depleted their insulin supply a few days ago due to a shortage of medication stemming from the ongoing war. Students must provide care to address the patient's immediate health needs. | Based on the history of present illness and the provided vital signs and physical examination findings, your task as an interprofessional team is to comprehensively assess and manage a 58-year-old patient admitted to the emergency department. |
| Case 2 | Posttraumatic stress disorder | A standardized patient presents with posttraumatic stress disorder. Students must provide care and psychological support to address immediate mental health needs. | Based on the history of present illness and the provided vital signs and physical examination findings, your task as an interprofessional team is to comprehensively assess and manage a Sara. |
| Case 3 | Chemical exposure | A standardized patient presents with symptoms of chemical exposure resulting from a secondary effect of the bombing, such as the release of hazardous substances. Students must conduct rapid assessments and initiate appropriate decontamination and treatment procedures. | Based on the history of present illness and the provided vital signs and physical examination findings, your task as an interprofessional team is to comprehensively assess and manage a 35-year-old patient. |
| Case 4 | Infectious disease outbreak | There is a concern for an infectious disease outbreak due to compromised sanitation and healthcare facilities. A standardized patient presents with symptoms of cholera, requiring students to provide care to address immediate health needs and implement infection control measures. | Upon reviewing the patient's history of present illness and considering the provided vital signs and physical examination findings, your interprofessional team is tasked with conducting a comprehensive assessment and developing a management plan for a 28-year-old patient. Additionally, your team must respond promptly to a sudden and widespread cholera outbreak affecting civilians. |

## **Supplementary Table 3. Qualitative insights into team performance, elucidating the rationale behind the overall global rating.**

| **Assessor number** | **Case title** | **Team number** | **Global rating scale** | **Why did you choose this rating for the overall global rating?** |
| --- | --- | --- | --- | --- |
| 1 | DKA | 1 | Below expectation | “Mainly because they did not address the concern at all. Many sides talk while one is talking to the PT. Focused on history gathering rather than the problem "lack of stock". Not equal contribution from all members.” |
| 1 | DKA | 2 | Above expectations | “Shared discussions with the patient. Some side talks. Not equal participation from all members.” |
| 1 | DKA | 3 | At expectations | “Trying to maintain active engagement of all members. Side talks very often and interrupting each other sometimes. Worked on finding an alternative for the PT.” |
| 1 | DKA | 4 | At expectations | “Team members were aware of the different backgrounds they're coming from and how each can help the patient, but their dynamics and communication as a "team" with the patient can be improved.” |
| 2 | DKA | 1 | At expectations | "Worked well together. Not much involvement from the patient makes more eye contact with answers as opposed to patient. Overall collaboration was seen, and perspective were considered but could have included patient more." |
| 2 | DKA | 2 | At expectations | "The team assigned roles immediately and did very well in establishing those roles. Each member contributed well and collaborated effectively in making the treatment plan. They also used differing ideas about the treatment plan, which they resolved effectively and in a respectful manner." |
| 2 | DKA | 3 | Above expectations | "Great introduction with addressing the patient. Effectively divided tasks among team members, and each team member made an effective contribution. Explained what they are doing to the patient well. Each member was engaged and addressed patient concerns well. Very impressed with this team." |
| 2 | DKA | 4 | At expectations | "Each member contributed by asking the patient questions and was engaged with the patient. Team members effectively engaged with patients and allocated roles well. Did not address patients' needs and concerns, just collected answers." |
| 3 | DKA | 5 | Below expectations | "The, although collaborated well, was not able to provide appropriate care for the patient." |
| 3 | DKA | 6 | Above expectations | "Very impressed, they put the patient at the center of the care. They stood up and approached the patient while each had time to ask relevant questions according to their profession and roles." |
| 3 | DKA | 7 | At expectations | "Good approach at the level of the teamwork, but the physician was at the spotlight. Patients' listening was not well, and team members were not focusing on patients' needs. They did generally well, but they could have done better." |
| 3 | DKA | 8 | Above expectations | "Team demonstrated high skills in functioning very well. I am impressed. They also provided appropriate care with the patient at the center of the care." |
| 4 | DKA | 5 | At expectations | "Only nursing student introduced her role. They asked good questions and were friendly with the patient. Empathy was not evident." |
| 4 | DKA | 6 | At expectations | "Need to work more collaboratively. Did the job individually with the patient. The physical therapy student was quiet in the team. Need to provide more empathy (Physical therapy student did great here)." |
| 4 | DKA | 7 | At expectations | "Did better individually. Team collaboration was not fully there. The doctor seemed to talk 90% of the time." |
| 4 | DKA | 8 | At expectations | "Some individuals introduced their roles (not all). Had good communication. Showed empathy." |
| 5 | PTSD | 1 | At expectations | None |
| 5 | PTSD | 2 | At expectations | None |
| 5 | PTSD | 3 | Above expectations | None |
| 5 | PTSD | 4 | Missing | None |
| 6 | PTSD | 1 | Above expectations | "They performed quite well and in collaboration. Overall, they were well-equipped with knowledge." |
| 6 | PTSD | 2 | At expectations | "They were performing really well. Were mostly responsive and interactive. Overall, they communicated well in trying to solve concerns." |
| 6 | PTSD | 3 | At expectations | "Overall, they showed good collaboration performance." |
| 6 | PTSD | 4 | Above expectations | "Overall, good team performance." |
| 7 | PTSD | 5 | At expectations | "Need to incorporate all professions. Talk to patient for plan and care." |
| 7 | PTSD | 6 | At expectations | "Professionals communicated well. Could have improvement with roles and responsibilities. Did well with patient/family-centered care." |
| 7 | PTSD | 7 | Below expectations | "Some interruptions between professionals. Need to give patients time to explain, some interruption to patients. Seek more patient input." |
| 7 | PTSD | 8 | Above expectations | "Good planning pre-discussion where all involved. Involved members and their responsibilities. Introduced names and professions. Empathetic communication, compassionate. Evaluating appropriate and involved patient. Please incorporate your responsibilities as a profession and mention other professions (i.e., nutritionist, psychologist). Looked for patients' thoughts on plan." |
| 8 | PTSD | 5 | Below expectations | "Functioned well as a team but can work on flow and interdisciplinary/interprofessional communication between members more." |
| 8 | PTSD | 6 | At expectations | "Could ask other team members for input. Dominated by medicine. Speaking about the plan as if the patient was not there at the end." |
| 8 | PTSD | 7 | Below expectations | "Lots of interruptions of team members. Great active listening when speaking to the patient. Positive affirmations provided to the patient. Could be more collaborative as a team. Try to be more relaxed from the beginning as a team. Lack of a common voice, all competing to speak, despite having a great plan. Breakdown in communication between the team members at the end of the exercise, which would affect patient care negatively. Lots of different messages provided to the patient." |
| 8 | PTSD | 8 | Above expectations | "Great team dynamics. Excellent sharing of goals and collaboration. Great inclusiveness." |
| 9 | Chemical exposure | 1 | Above expectations | "Harmony while team is functioning. Active listening. Patient is comfortable. The team is very good." |
| 9 | Chemical exposure | 2 | Below expectations | "They were discussing among themselves, not engaging the patient. Using medical terms with the patient. Not identifying their roles. Side talks. Conflict between two members (high tone)." |
| 9 | Chemical exposure | 3 | At expectations | "It was a functional team. They worked perfectly even though there were 2 missing members. However, they did not introduce themselves to the patient and specify roles." |
| 9 | Chemical exposure | 4 | At expectations | None |
| 10 | Chemical exposure | 1 | Above expectations | "The group was very functional and calm, working together in a harmonized way." |
| 10 | Chemical exposure | 2 | Below expectations | "They were focused on searching, arguing, and having side discussions, more than interacting with the patient." |
| 10 | Chemical exposure | 3 | At expectations | "It was a good functional team, but I had more expectations. They did not introduce themselves and did not clarify their roles to the patient." |
| 10 | Chemical exposure | 4 | At expectations | None |
| 11 | Chemical exposure | 5 | At expectations | "Had contributions but not all members were active. The physician was very active and guided the entire group." |
| 11 | Chemical exposure | 6 | Below expectations | "Lack of team communication. The physician was not up to the level expected." |
| 11 | Chemical exposure | 7 | At expectations | "The team performed well. They could catch up and manage the situation. They had good communication and covered almost all aspects they must deal with. They could not think that the patient is not from or may not be from a medical background. All over very good." |
| 11 | Chemical exposure | 8 | At expectations | "Had reasonable contributions. Tried to cover all above criteria. The physician was not up to the level as expected. The rest was very good. The nurses are excellent." |
| 13 | Infectious disease outbreak | 1 | Above expectations | "Care plan is given to the patient, but it was confusing. Conflicting plan: advising the patient to take precautions to not contaminate her sister at home and then telling her that she will be admitted to the hospital. The SP had to ask a lot of questions to clarify the plan. Team was very confident and functioned closely with the hospital." |
| 13 | Infectious disease outbreak | 2 | Below expectations | "The team introduced themselves to the SP (name of the professional). The group consisted of 4 (they excluded one student in the way of seating and leading the case). The introduction was not engaging. Replicated information. Team did not agree on a clear intervention plan, and SP was confused. Team members had conflicting thoughts." |
| 13 | Infectious disease outbreak | 3 | Above expectations | "Team did not introduce team members. Excellent interventions. However, 1 student did not interfere with very little input. Decided care plan. Team collaborated effectively together." |
| 13 | Infectious disease outbreak | 4 | At expectations | "Group was more focused on seeking information from the SP. Questions were not organized. No intervention was given." |
| 14 | Infectious disease outbreak | 1 | Above expectations | "They shared responsibilities and had defined roles. They did not have a clear response plan with regard to admission until the patient asked, but they explained it very clearly afterwards." |
| 14 | Infectious disease outbreak | 2 | At expectations | "Discussed the response plan with the patient. Discussed the side effects of the medications and ensured the patient understood the use/benefits/risks of the medications. They did not know how to resolve the conflicts or have a unified response." |
| 14 | Infectious disease outbreak | 3 | Above expectations | "Introduced the team. Collaborated perfectly. Shared information effectively with the patient. Explained the scientific information simply. Only 2 members were leading the discussion; one member (nurse) was mostly silent, probably because 2 nursing students were there." |
| 14 | Infectious disease outbreak | 4 | At expectations | "They collaborated properly and effectively. Yet, they did not define responsibilities and they did not introduce themselves. Also, they did not share solutions with the patient." |

Abbreviations: DKA, Diabetic ketoacidosis; PTSD, Posttraumatic stress disorder.
